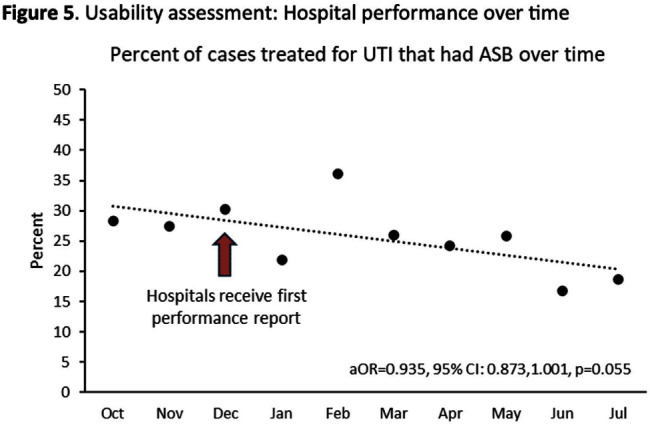# Validation and Use of a Measure in Critical Access Hospitals to Reduce Treatment of Asymptomatic Bacteriuria

**DOI:** 10.1017/ash.2024.215

**Published:** 2024-09-16

**Authors:** Hannah Imlay, Claire Ciarkowski, Zahra Kassamali Escobar, Chloe Bryson-Cahn, Adam Hersh, Natalia Martinez-Paz, Whitney Hartlage, Jeannie Chan, Hannah Hardin, Andrea White, Chaorong Wu, Valerie Vaughn

**Affiliations:** University of Utah; University of Washington; Veteran’s Affairs Salt Lake City Health Care System; Harborview Medical Center; Division of General Internal Medicine; University of Utah School of Medicine

## Abstract

**Background:** Inappropriate diagnosis and treatment of urinary tract infections (UTI) contributes to antibiotic overuse. The Inappropriate Diagnosis of UTI (ID-UTI) measure—which quantifies the percentage of treated bacteriuria that is asymptomatic (ASB) using a standard definition of UTI—has been validated in acute care hospitals, but not in critical access hospitals (CAHs) where resources differ. **Methods:** From October 2022-July 2023, ten CAHs participated in a program to reduce ASB treatment, including education, coaching, and hospital feedback using the ID-UTI measure. Our primary aim was to assess performance characteristics of the ID-UTI standard definition and measure in CAHs (Figure 1). Non-physician abstractors from each CAH submitted clinical data (e.g., signs/symptoms) via REDCap for consecutive adult patients who were admitted or discharged from the emergency department and received antibiotics for bacteriuria. The case abstraction goal for each CAH was 5-6 cases/month. To assess feasibility, we evaluated the ability of each CAH to achieve goal case abstractions. To assess validity and reliability of the ID-UTI standard definition, two physicians reviewed 10% of submitted cases using deidentified patient notes and assessed agreement with the standard ID-UTI definition and consensus clinical opinion. Based on submitted data, we provided bi-monthly feedback reports to CAHs including the hospital-level ID-UTI measure to benchmark progress and for peer comparison. We measured monthly change in the ID-UTI measure using a mixed-effects logistic regression model (Figure 1, Figure 2). **Results:** Among 10 CAHs, 4 (40%) submitted >59 cases over 10 months (goal) while 3 (30%) submitted >35 cases (secondary goal). Physician reviewers assessed 9.5% (58/608) of cases. Utilizing the ID-UTI standard definition, there was high agreement (93%) in ASB vs UTI designation between each physician reviewer and the CAH’s REDCap assessment (Figure 3). Compared to clinical opinion, the ID-UTI standard definition identified 48% (16/33) of ASB cases and 100% (25/25) of UTI cases (Figure 4). Over the program, the percentage of cases treated for UTI that were ASB decreased from 28.4% (range 0-63%) to 18.6% (range, 0-33%; p=0.055) (Figure 5). **Conclusions:** Case abstraction with use of the ID-UTI measure was feasible and reliable to implement with modifications for CAHs. Data collection by untrained staff was as reliable as physician adjudication. Though the ID-UTI standard definition undercounted ASB cases (low sensitivity), cases reported as ASB were always ASB when adjudicated (high specificity). The program, including performance feedback using the ID-UTI measure, was associated with a trend toward lower treatment of ASB.

**Figure:**
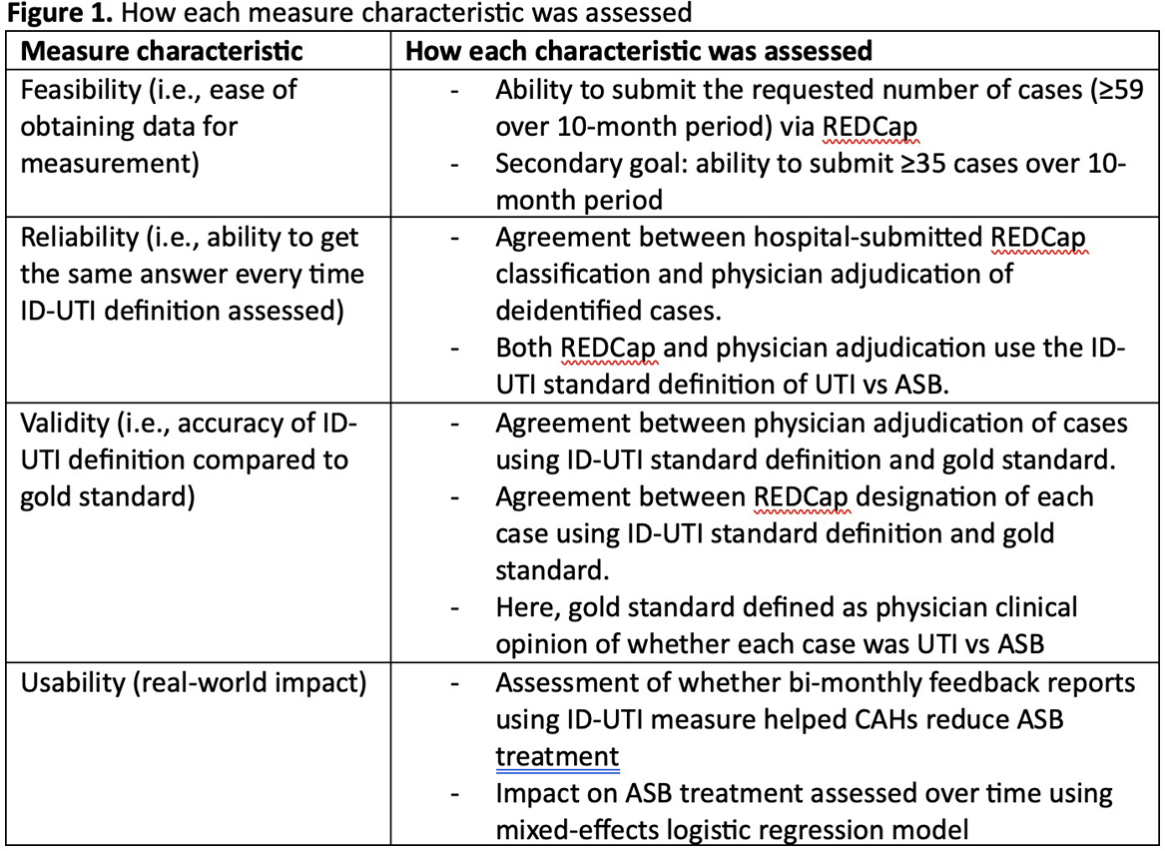

**Figure:**
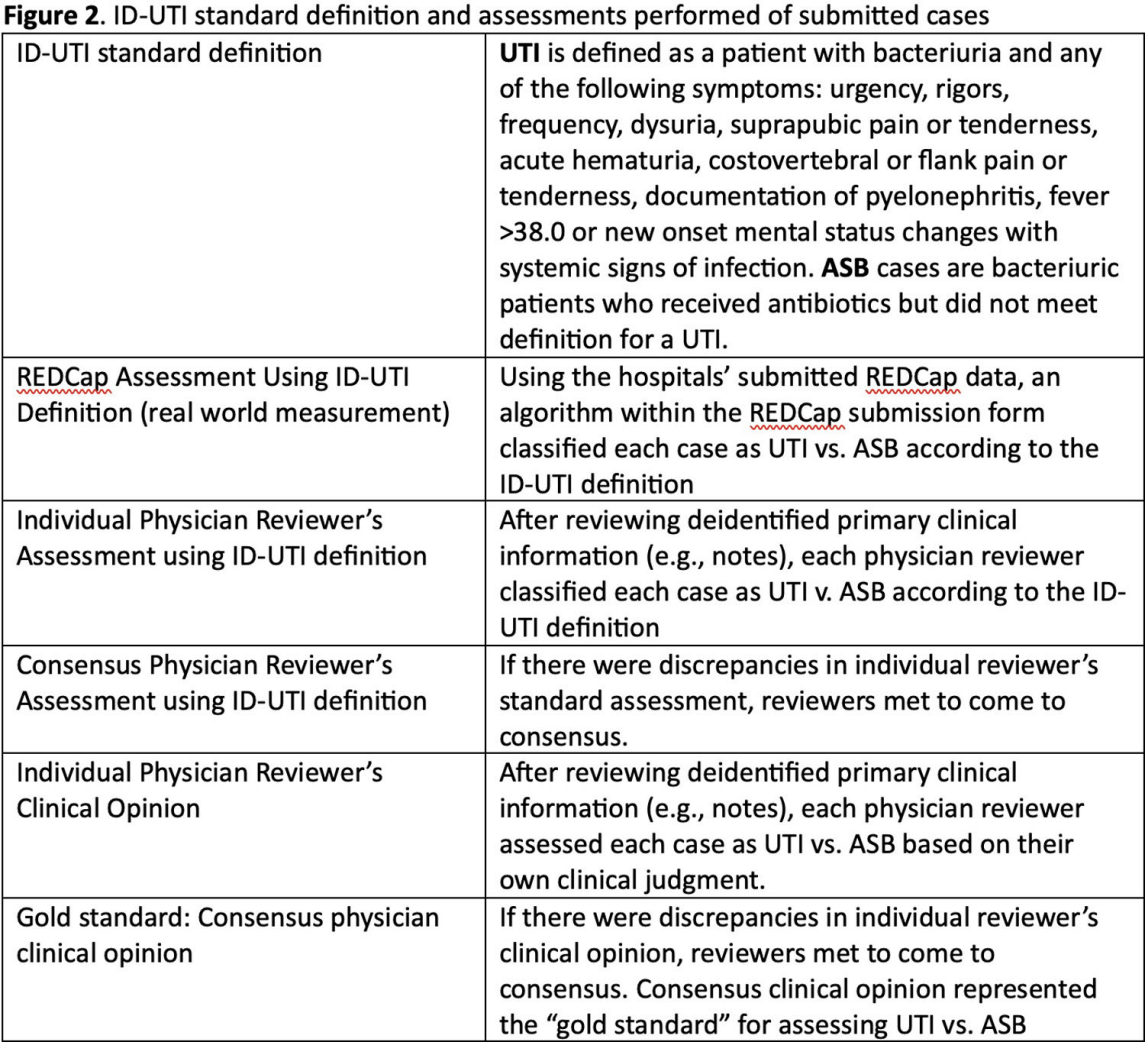

**Figure:**
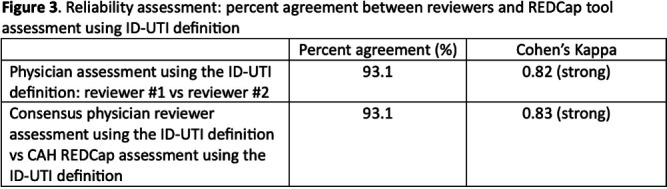

**Figure:**
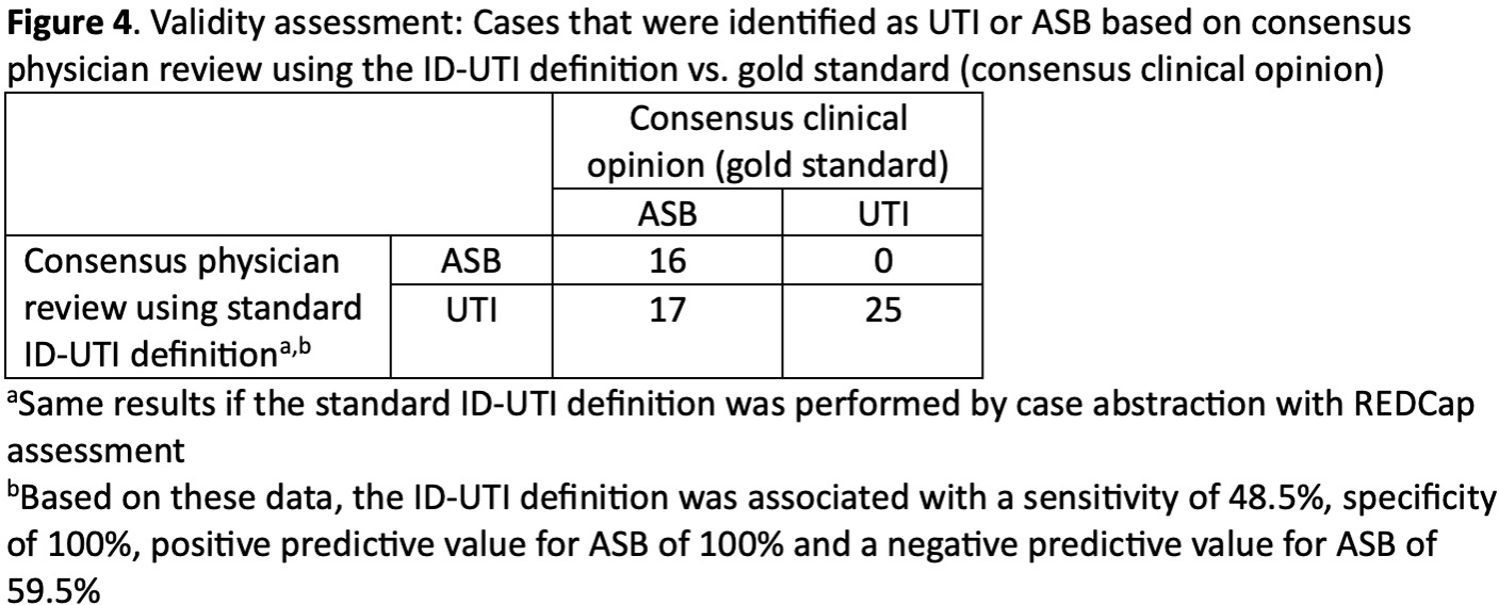

**Figure:**